# Fracture Risk and Use of Angiotensin-Converting Enzyme Inhibitors or Angiotensin II Receptor Blockers

**DOI:** 10.1007/s00223-022-01004-9

**Published:** 2022-07-14

**Authors:** Kara L. Holloway-Kew, Amelia G. Betson, Kara B. Anderson, Filip Sepetavc, James Gaston, Mark A. Kotowicz, Wan-Hui Liao, Maciej Henneberg, Julie A. Pasco

**Affiliations:** 1 Deakin University, IMPACT – the Institute for Mental and Physical Health and Clinical Translation, School of Medicine, Health Education and Research Building, Level 3 (Barwon Health), PO Box 281, Geelong, VIC 3220 Australia; 2 Barwon Health, Geelong, Australia; 3 Department of Medicine, The University of Melbourne – Western Health, St Albans, Australia; 4 Department of Medical Education, Taipei Veterans General Hospital, Taipei, Taiwan; 5 Biological and Comparative Anatomy Research Unit, Adelaide Medical School, University of Adelaide, Adelaide, Australia; 6 Institute of Evolutionary Medicine, University of Zurich, Zurich, Switzerland; 7 Department of Archaeology, Flinders University, Adelaide, Australia; 8 Department of Epidemiology and Preventive Medicine, Monash University, Prahran, Australia; 9 Department of Internal Medicine, Taipei City Hospital Yangming Branch, Taipei, Taiwan

**Keywords:** Angiotensin converting enzyme inhibitors, Angiotensin II receptor blockers, Fracture risk

## Abstract

Medications used to treat hypertension may affect fracture risk. This study investigated fracture risk for users of angiotensin converting enzyme inhibitors (ACEI) or angiotensin II receptor blockers (ARB). Participants (899 men, median age 70.3 yr (59.9–79.1), range 50.0–96.6 yr; 574 women, median age 65.5 yr (58.1–75.4), range 50.1–94.6 yr) were from the Geelong Osteoporosis Study. Medication use was self-reported and incident fractures were ascertained using radiological reports. Bone mineral density (BMD) was measured at the femoral neck. Participants were divided into four groups: (1) non-users without hypertension, (2) non-users with hypertension, (3) ACEI users and (4) ARB users. Dosage was calculated using the defined daily dose (DDD) criteria. Participants were followed from date of visit to first fracture, death or 31 December 2016, whichever occurred first. Cox proportional hazards models were used for analyses. At least one incident fracture was sustained by 156 men and 135 women over a median(IQR) of 11.5(6.2–13.2) and 10.9(6.3–11.6) years of follow-up, respectively. In unadjusted analyses, compared to non-users without hypertension, men in all three other groups had a higher risk of fracture (Hazard Ratio (HR, 95%CI) 1.54, 1.00–2.37; 1.90, 1.18–3.05; 2.15, 1.26–3.66), for non-users with hypertension, ACEI and ARB users, respectively). Following adjustment for age, prior fracture and BMD, these associations became non-significant. A dose effect for ARB use was observed; men using lower doses had a higher risk of fracture than non-users without hypertension, in both unadjusted (2.66, 1.34–5.29) and adjusted (2.03, 1.01–4.08) analyses, but this association was not observed at higher doses. For women, unadjusted analyses showed a higher risk for ACEI users compared to non-users without hypertension (1.74, 1.07–2.83). This was explained after adjustment for age, alcohol consumption, prior fracture and BMD (1.28, 0.74–2.22). No other differences were observed. In men, lower dose (0 < DDD ≤ 1) ARB use was associated with an increased risk of fracture. ACEI or ARB use was not associated with increased risk of incident fracture in women. These findings may be important for antihypertensive treatment decisions in individuals with a high risk of fracture.

## Introduction

Osteoporosis and hypertension are both common conditions in older adults and share risk factors such as older age, low physical activity, poor diet and smoking [1–3]. Both osteoporosis and hypertension can affect the risk of future fracture.

Osteoporosis is a systemic condition that results in an increased risk of fracture through a reduction in the quantity and quality of bone, such that even low trauma can result in a fracture. Although hypertension is not considered an independent risk factor for fracture, it has been suggested to be associated with an increased risk. A potential mechanism for this has been described, whereby individuals with hypertension have a net loss of calcium due to increased urinary excretion as well as reduced absorption [4, 5]. This can then lead to increased activation of parathyroid tissue and a net loss of calcium from the bones [5]. Additionally, hypertension may result in a higher level of oxidative stress, which could also lead to poorer bone health [6]. Some medications used to lower blood pressure can result in orthostatic hypotension, particularly when first prescribed and this can increase the risk of falls which can lead to fracture [5]. There are several different classes of medication used to treat hypertension and these include angiotensin converting enzyme inhibitors (ACEIs) and angiotensin II receptor blockers (ARBs), which function by affecting the renin aldosterone angiotensin system (RAAS). These medications may affect fracture risk, as components of the RAAS are also present on bone cells, specifically osteoclasts and osteoblasts [7]. Activation of this system on bone cells results in an increased bone resorption and decreased bone formation, which could lead to poorer bone quality and quantity [8, 9].

Several studies have examined associations between ACEI and/or ARB use and fracture outcomes. ARB use has consistently been associated with lower or similar fracture rates compared to non-users [10–16]. However, ACEI use has been associated with lower fracture rates [15–17], no difference in fracture rates [11, 13, 14] and higher fracture rates [10, 18] compared to non-users. Part of the reason for these conflicting results is the heterogeneity amongst studies, as described in a meta-analysis by Cheng et al. [19]. This meta-analysis of observational studies indicated that age was the most important source of heterogeneity and that ACEI use was associated with an increased risk of fracture, which was more pronounced in those aged ≥ 65 years. Another important contribution to the observed heterogeneity was that some studies did not account for other important variables such as weight.

It has been estimated that after the age of 50 years, one in three women and one in five men will suffer an osteoporotic fracture in their remaining lifetime [20]. This age group is also at risk of hypertension; the National Heart Foundation of Australia guidelines [21] recommend that primary prevention for cardiovascular disease is targeted at individuals aged ≥ 45 years. Men and women may be differently affected by hypertension; on average, women have a lower blood pressure until the age of menopause, after which the average blood pressure in women will surpass that in men [22, 23]. Additionally, men may have a reduced response to RAAS inhibition compared to women [24, 25] and therefore use of antihypertensive medication may produce different results by sex. It is likely that both men and women in the age range of ≥ 50 years will be at elevated risk for fracture and may use antihypertensive medications. Therefore, the aim of this study was to investigate the association between ACEI or ARB use and longitudinal fracture risk in this age group of men and women, adjusting for potential confounders.

## Methods

### Participants

Participants for these retrospective cohort analyses were drawn from the Geelong Osteoporosis Study, which included randomly selected residents of the Barwon Statistical Division, located in south-eastern Australia. Participants were drawn from the electoral roll, to which enrolment is compulsory in Australia, and age stratified to capture the full adult age-range. They have provided a wide range of data including but not exclusive to questionnaires, anthropometry and DXA scans. Baseline assessments for women occurred in 1993–1997 and for men in 2001–2006, with participation of 77% and 67%, respectively. Participants have returned for follow-up visits every few years. Further details of the study have been described previously [26]. Data for this study are derived from the baseline visit for men (2001–2006) and 10-year follow-up for women (2004–2008). These follow-up visits were selected as they occurred after the introduction of ARB medications in Australia (1997) [27]. According to Australian Pharmaceutical Benefits Scheme data [28], ARB use was initially low (27,627 prescriptions written in 1997), however this increased over time and during the study period (2001–2008) the number of prescriptions plateaued; 5,421,146 in 2001, 9,446,943 in 2004 and 9,527,533 in 2007. The increasing and widespread use of ARB medication by the time of the study period shows that ARB medications had been available for a sufficient time following their introduction in 1997 and therefore, it is not expected that there would be any significant effect on the study results.

All data presented focussed on the baseline visit for men and 10-year follow-up for women, however it should be noted that at the 10-year follow-up for women, eligible responders (73.5%) showed a lower median baseline age than non-responders (67.2 vs 83.4 years, *p* < 0.001), likely due to mortality being higher in older age groups. Responders at this follow-up also were taller (160.7 vs 158.6 cm) at baseline than their non-responding peers, however, weight was not different (70.0 vs 68.6 kg, *p* = 0.214). Participants aged ≥ 50 years were included in this study, resulting in 930 men and 578 women of an appropriate age to be eligible for analyses.

**Fig. 1:**
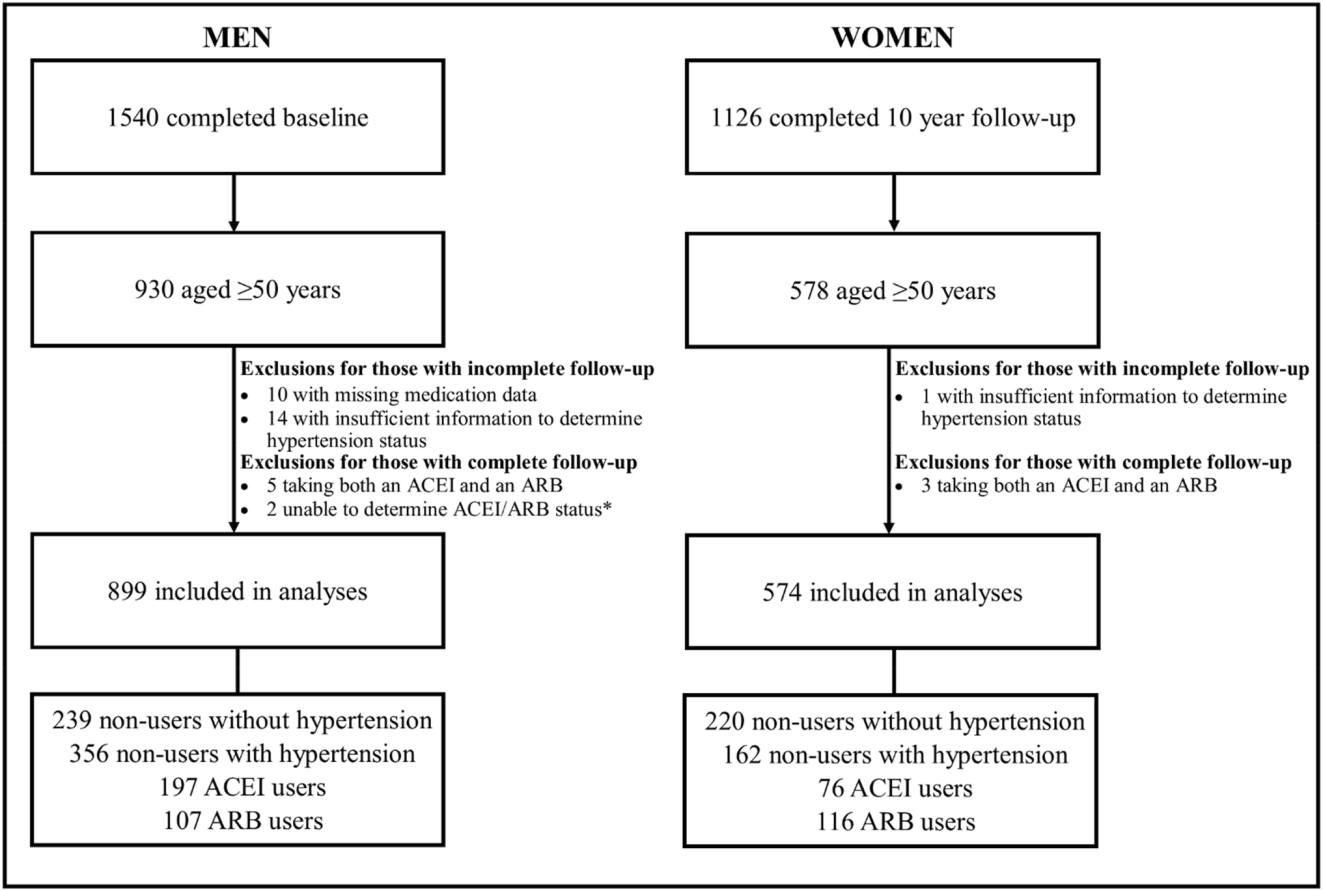
Participant flow chart for men and women in this study. *ACEI* angiotensin converting enzyme inhibitor, *ARB* angiotensin II receptor blocker. *Two men were participating in a clinical trial that involved the use of either an ACEI/ARB medication or placebo

Of the 930 eligible men, 31 were excluded: 10 were excluded due to missing medication data, five were taking both an ACEI and an ARB and 14 due to insufficient information to determine hypertension status. Two other men were excluded due to inability to determine ACEI/ARB use status, as they were included in a clinical trial which involved the use of an ACEI/ARB medication or placebo. Of the 578 eligible women, four were excluded: three were taking both an ACEI and ARB medication and one had insufficient information to determine hypertension status. This left 899 men and 574 women included in the analyses. A participant flow chart is presented in Fig. 1.

Barwon Health Human Research Ethics Committee approved the study (projects 92/01 and 00/56).

### Medication Use

Current medication use at a single time point (time of study visit) was obtained by self-report, including dose, frequency and date started. Participants were encouraged to bring their medication containers when attending appointments with the research team. ACEI and ARB use was determined, as well as use of glucocorticoids, other antihypertensive medications (including thiazide diuretics) and statins. Use of medications known to have a positive effect on bone were also identified by examination of participant questionnaires, specifically including bisphosphonates, calcium and/or vitamin D supplements, and for women, hormone replacement therapy.

Since different types of ACEI and ARB medications use different doses, these were converted using the defined daily dose (DDD) as described by the World Health Organization Collaborating Centre for Drug Statistics Methodology [29]. It allows standardisation of doses between different drugs. The DDD is calculated by the following formula: dose of drug taken/dose of drug in a DDD = number of DDDs. For this study, ‘low dose’ was considered as 0 < DDD ≤ 1 and ‘high dose’ as DDD > 1.0.

### Incident Fracture Ascertainment

Incident fractures were ascertained using a computerised keyword search across reports from all radiological imaging centres within the region independent to participant involvement in study visits [2, 30]. Upon review, only clearly defined fractures were included. Where a report was listed as “suggestive” or “possible” for fracture, these were included only when a subsequent report was available for confirmation. This method of fracture ascertainment has been validated [31]. Fractures of the face, skull, fingers and toes were excluded, as well as those occurring from pathology or high trauma, as indicated in the report. Cause of fracture was coded via ICD-9 codes and included codes 885 (fall on same level from slipping, tripping or stumbling), 886 (fall on same level from collision, pushing or shoving, by or with other person), 887 (fracture, cause unspecified), 888 (other and unspecified fall) and 927 (overexertion and strenuous movements).

### Other Variables

Weight and height were measured using electronic scales and a Harpenden wall-mounted stadiometer to the nearest 0.1 kg and 0.001 m, respectively. Body mass index (BMI) was calculated as weight/height (kg/m^2^). Blood pressure (mmHg) was measured in a seated position using an automated device (Takeda Medical UA-751). Femoral neck BMD (g/cm^2^) was measured for women using a GE-Prodigy (Prodigy; GE Lunar, Madison, WI, USA) dual-energy X-ray absorptiometry densitometer. The GE-Prodigy was also used for 513 men and a Lunar DPX-L (Lunar; Madison, WI, USA) was used for 352 men. No differences were observed in cross-calibration testing of these two machines [26]. Thirty-four men did not complete a dual-energy X-ray absorptiometry scan at their visit but provided sufficient information regarding medication use to be included in this study.

The following data were self-reported by participants from questionnaires. Alcohol consumption was determined using a food frequency questionnaire developed by the Victorian Cancer Council [32]. High-alcohol consumption was considered as ≥ 30 g of alcohol per day. Information on falls was categorised as 0 or ≥ 1 over the previous 12 months. Physical activity was self-reported using a seven point scale as previously described [26] and included: very active, active, sedentary, limited, inactive, chair or bedridden and bedfast. These were then categorised into “high” physical activity, including very active and active, and “low” physical activity including the remaining groups. Smoking status was categorised as currently smoking or not. Fractures occurring prior to the baseline visit for men and the 10-year follow-up for women were identified by self-report and confirmed using radiological reports where possible.

Charlson Comorbidity Scores [33] were calculated for each participant using a combination of self-reported, measured and linkage data. Non self-reported data included ascertainment of diabetes status, where participants were classified as having diabetes if they had fasting plasma glucose ≥ 7.0 mmol/L (126 mg/dL), self-reported diabetes and/or used antihyperglycemic medications. All data on cancer from 1986 onwards were obtained from linkage with the Victorian Cancer Registry. Rheumatoid arthritis (considered under connective tissue disease) was self-reported and confirmed using medication and medical record data from the University Hospital Geelong (UHG). The presence of acquired immunodeficiency syndrome was determined by examining self-reported medications, followed by UHG medical records. The remaining items were obtained by self-report. UHG medical records were also used to confirm the presence of these medical conditions. Data for congestive heart failure and peripheral vascular disease were not available for men at the baseline visit, and the Charlson Comorbidity Score was calculated without these conditions.

The Index of Relative Socio-Economic Advantage and Disadvantage (IRSAD) was also determined for each participant and is an indicator of socioeconomic status (SES). The IRSAD accounts for both disadvantage and advantage; it includes income and occupation (unskilled employment to professional positions) [34]. A higher score represents a more advantaged area, while a lower score indicates a more disadvantaged area. These scores were divided into quintiles, where quintile 1 represents the most disadvantaged and quintile 5 the most advantaged.

### Statistical Analysis

Statistical analyses were conducted separately for men and women. The participants were then divided into four groups: (1) non-users (of ACEI or ARB medications) without hypertension, (2) non-users with hypertension, (3) ACEI users and (4) ARB users. Hypertension for group 2) was categorised as self-reported hypertension, use of antihypertensive medication except ACEI or ARB, systolic blood pressure ≥ 140 mmHg and/or diastolic blood pressure ≥ 90 mmHg. Using these criteria, it may have been possible to capture participants with either controlled or uncontrolled hypertension, however, in this study there were no participants with uncontrolled hypertension. All participants categorised as having hypertension were also taking an antihypertensive agent. In analyses, non-users without hypertension (group 1) were set at the referent group. These groups were created because hypertension status has previously been reported to affect fracture risk [4].

The normality of continuous variables was tested using the Shapiro–Wilk test. Height, weight, BMI and femoral neck BMD were normal; these were described using means and standard deviations (SD). The remaining variables were described using medians and interquartile range (IQR). Differences between groups were assessed using ANOVA for height and femoral neck BMD and Kruskal–Wallis for the other variables. Categorical data were described using *n*(%) and differences assessed using Chi-squared tests. Dunn and Tukey tests were also used to further investigate differences in continuous variables between groups, employing a *p* value < 0.01.

Participants were followed from baseline or 10-year follow-up (men and women, respectively) to date of first fracture, date of death or the end of the study period (31 December 2016), whichever occurred first. Kaplan–Meier plots were generated, and Log-rank tests were used to assess unadjusted fracture risk across the groups. Cox proportional hazard models were used for multivariable (adjusted) survival analysis including the following variables: age, weight, height, alcohol consumption, falls, physical activity, smoking status, prior fracture, statin use, thiazide diuretic use, glucocorticoid use, use of medications with a positive effect on bone, Charlson Comorbidity Score, SES and femoral neck BMD. These variables were first tested in bivariate analyses and those that were significant (*p* < 0.05) were included in the final multivariable model. Those variables that remained significant in the final model were retained. This was completed independently for men and women, resulting in different variables being included in the final multivariable models. The effect of ACEI or ARB dose was also explored, and there was evidence of a dose effect for ARBs in men. Thus an additional analysis was performed for men, stratifying ARB dose using “high” and “low” DDD categories defined above. No evidence of a dose effect for either ACEI or ARB use was observed in women and no additional analyses were performed. Hazard ratios (HRs) and 95% confidence intervals (CIs) from the Cox model were reported. Proportional hazard model assumptions were tested and all models met the assumptions. Interaction terms were also tested and none were identified.

Analyses were completed using Minitab (Minitab, version 18, State College, PA, USA) and STATA (Version 15.1. StataCorp. 2017. Stata Statistical Software: Release 15. College Station, TX: StataCorp LLC).

## Results

### Men

Of 899 men, 197 (21.9%) used an ACEI medication, 107 (11.9%) used an ARB medication and 595 (66.2%) used neither an ACEI or ARB medication (“non-users”). Among non-users, 356 men had hypertension and 239 did not.

#### Descriptive Characteristics

Compared to non-users without hypertension, men in the other three groups were older, shorter, had a higher BMI, higher blood pressure, were more likely to use statins or thiazide diuretics and had a higher Charlson Comorbidity Score (Table 1). A difference was detected between the groups for SES, where men in the non-users without hypertension group were overrepresented in quintiles 4 and 5 compared to the other three groups. Men taking ACEI or ARB medications were more likely to have lower physical activity than the other two groups, whereas men taking an ARB medication had a greater weight than the other three groups.

**Table 1:** Descriptive statistics for men and women stratified by use of angiotensin converting enzyme inhibitors (ACEI) or angiotensin receptor blockers (ARB)

Men	Non-users without hypertension (*n* = 239)	Non-users with hypertension (*n* = 356)	ACEI (*n* = 197)	ARB (*n* = 107)	*p* value
Age (yr)	61.6 (54.3–72.5), range 50.2–96.6	71.2 (61.8–80.2), range 50.0–93.6	75.4 (66.3–82.2), range 50.5–92.7	73.4 (61.9–80.6), range 50.2–93.5	< 0.001
Weight (kg)	80.6 ± 13.1	81.8 ± 13.9	82.7 ± 14.1	85.3 ± 13.4	0.034
Height (cm)	174.1 ± 7.1	172.3 ± 6.6	171.8 ± 6.9	172.3 ± 5.6	0.002
Body mass index (kg/m^2^)	26.5 ± 3.5	27.5 ± 4.2	28.0 ± 4.3	28.7 ± 4.2	< 0.001
Systolic blood pressure (mmHg)	126 (120–132)	145 (135–157)	138 (128–154)	140 (130–154)	< 0.001
Diastolic blood pressure (mmHg)	81 (76–85)	91 (81–99)	84 (75–92)	84 (77–93)	< 0.001
High alcohol consumption	49 (20.5)	74 (20.8)	38 (19.3)	25 (23.4)	0.897
Falls (one or more over past 12 months)	65 (27.2)	113 (31.7)	67 (34.0)	31 (29.0)	0.426
Low physical activity	60 (25.1)	98 (27.5)	70 (35.5)	39 (36.5)	0.033
Smoking	20 (8.4)	40 (11.2)	14 (7.1)	9 (8.4)	0.381
Prior fracture	40 (16.7)	74 (20.8)	28 (14.2)	25 (23.4)	0.123
Statin use	24 (10.0)	74 (20.8)	87 (44.2)	35 (32.7)	< 0.001
Thiazide diuretic use	0 (0.0)	7 (2.0)	16 (8.1)	20 (18.7)	< 0.001
Antihypertension medication use^a^	0 (0.0)	127 (35.7)	119 (60.4)	58 (54.2)	< 0.001
Glucocorticoid use	4 (1.7)	12 (3.4)	7 (3.6)	3 (2.8)	**–**
Medications with a positive effect on bone^b^	7 (2.9)	20 (5.6)	12 (6.1)	6 (5.6)	0.746
Charlson Comorbidity Score	0 (0–1)	0 (0–1)	1 (0–2)	0 (0–1)	< 0.001
SES^c^					0.044
Quintile 1 (most disadvantaged)	32 (13.4)	75 (21.1)	44 (22.3)	22 (20.6)	
Quintile 2	47 (19.7)	76 (21.4)	51 (25.9)	21 (19.6)	
Quintile 3	43 (18.0)	82 (23.0)	31 (15.7)	20 (18.7)	
Quintile 4	57 (24.0)	60 (16.9)	30 (15.2)	20 (18.7)	
Quintile 5 (most advantaged)	60 (25.1)	63 (17.7)	41 (20.8)	24 (22.4)	
Femoral neck BMD (g/cm^2^)	0.954 ± 0.128	0.947 ± 0.150	0.929 ± 0.148	0.944 ± 0.153	0.380
Any incident fracture	31 (13.0)	63 (17.7)	38 (19.3)	24 (22.4)	0.487
Incident fracture rate per 100,000 person years follow-up (95%CI)	1.18 (0.80–1.67)	1.80 (1.39–2.30)	2.21 (1.57–3.01)	2.51 (1.62–3.71)	< 0.001

Women	Non-users without hypertension (*n* = 220)	Non-users with hypertension (*n* = 162)	ACEI (*n* = 76)	ARB (*n* = 116)	*p* value
Age (yr)	60.1 (55.2–67.2), range 50.1–92.2	69.8 (61.1–78.1), range 50.3–94.6	71.8 (63.0–79.2), range 50.7–92.2	68.8 (60.4–77.7), range 50.2–92.7	< 0.001
Weight (kg)	69.3 ± 13.3	73.3 ± 14.7	72.3 ± 17.0	76.6 ± 16.3	< 0.001
Height (cm)	161.4 ± 6.2	159.0 ± 6.9	158.2 ± 6.2	159.1 ± 6.4	< 0.001
Body mass index (kg/m^2^)	26.6 ± 4.9	29.0 ± 5.6	28.8 ± 6.6	30.2 ± 6.1	< 0.001
Systolic blood pressure (mmHg)	123 (114–132)	141 (130–151)	136 (122–147)	137 (127–146)	< 0.001
Diastolic blood pressure (mmHg)	76 (71–80)	82 (76–89)	76 (69–85)	78 (72–85)	< 0.001
High alcohol consumption	14 (6.4)	6 (3.7)	2 (2.6)	3 (2.6)	–
Falls (one or more over past 12 months)	60 (27.3)	53 (32.7)	29 (38.2)	36 (31.0)	0.361
Low physical activity	40 (18.2)	58 (35.8)	35 (46.1)	47 (40.5)	< 0.001
Smoking	27 (12.3)	9 (5.6)	6 (7.9)	8 (6.9)	0.099
Prior fracture	32 (14.5)	37 (22.8)	21 (27.6)	24 (20.7)	0.058
Statin use	23 (10.5)	27 (16.7)	23 (30.3)	40 (34.5)	< 0.001
Thiazide diuretic use	0 (0.0)	7 (4.3)	10 (13.2)	22 (19.0)	< 0.001
Antihypertension medication use^a^	0 (0.0)	87 (53.7)	52 (68.4)	61 (52.6)	< 0.001
Glucocorticoid use	6 (2.7)	4 (2.5)	2 (2.6)	1 (0.9)	–
Medications with a positive effect on bone^b^	35 (15.9)	15 (9.3)	21 (27.6)	18 (15.5)	0.004
Charlson Comorbidity Score	0 (0–0)	0 (0–1)	1 (0–1)	0 (0–1)	< 0.001
SES^c^					0.272
Quintile 1 (most disadvantaged)	29 (13.2)	34 (21.0)	14 (18.4)	22 (19.0)	
Quintile 2	44 (20.0)	30 (18.5)	13 (17.1)	19 (16.4)	
Quintile 3	48 (21.8)	46 (28.4)	20 (26.3)	28 (24.1)	
Quintile 4	45 (20.5)	22 (13.6)	14 (18.4)	29 (25.0)	
Quintile 5 (most advantaged)	54 (24.6)	30 (18.5)	15 (19.7)	18 (15.5)	
Femoral neck BMD (g/cm^2^)	0.875 ± 0.136	0.847 ± 0.160	0.829 ± 0.160	0.885 ± 0.172	0.041
Any incident fracture	47 (21.4)	36 (22.2)	25 (32.9)	27 (23.3)	0.495
Incident fracture rate per 100,000 person years follow-up (95%CI)	2.26 (1.66–2.99)	2.58 (1.81–3.55)	4.05 (2.64–5.92)	2.55 (1.69–3.69)	< 0.001

Data presented as mean ± SD, median(IQR) or n(%)

^a^Includes calcium channel blockers, beta blockers and diuretics

^b^Includes bisphosphonates, calcium and/or vitamin D supplements and hormone replacement therapy

^c^Socioeconomic status

Missing data: Men: weight *n* = 33, height *n* = 33, body mass index *n* = 33, blood pressure *n* = 91, alcohol consumption *n* = 49, falls *n* = 2, statin use *n* = 161, glucocorticoid use *n* = 161, medications with a positive effect on bone *n* = 161, femoral neck bone mineral density *n* = 65. Women: weight *n* = 16, height *n* = 15, body mass index *n* = 16, blood pressure *n* = 59, alcohol consumption *n* = 21, falls *n* = 5, physical activity *n* = 4, smoking *n* = 4, prior fracture *n* = 2, glucocorticoid use *n* = 3, medications with a positive effect on bone *n* = 3, Charlson Comorbidity Index *n* = 4, femoral neck bone mineral density *n* = 41

#### Incident Fracture risk

During 8808 person-years of follow-up (median, IQR: 11.5, 6.2–13.2 years), 156 men sustained at least one fracture. This corresponded to a rate of 1.77 (95% CI 1.51–2.07) fractures per 100,000 person-years of follow-up. These included 70 vertebra, 19 hip, 18 rib, 15 forearm/wrist, 7 ankle, 6 humerus, 4 foot, 4 pelvis, 3 clavicle, 3 femur, 3 hand, 3 tibia/fibula and 1 patella fracture. The duration of follow-up for those with and without fracture were (median, IQR) 5.4 years (2.7–9.0) and 12.4 years (8.0–13.3), respectively.

**Fig. 2:**
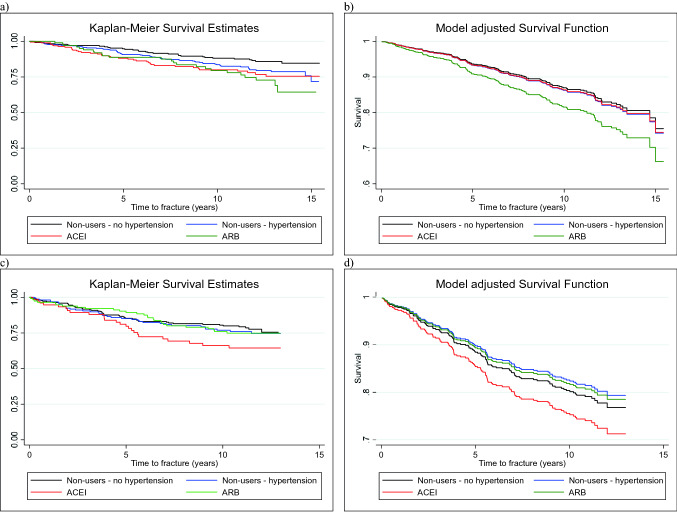
Unadjusted and adjusted cumulative survival functions for angiotensin converting enzyme inhibitor (ACEI) or angiotensin receptor blocker (ARB) use versus fracture survival time. Panels show: **a** Men, unadjusted, **b** Men, adjusted, **c** Women, unadjusted and **d** Women, adjusted

Figure 2a shows the Kaplan–Meier plot for incident fracture risk in each of the groups. In unadjusted analyses, compared to non-users without hypertension, all three of the other groups (non-users with hypertension, ACEI users and ARB users) had a higher risk of fracture (Table 2). In analyses adjusted for age, prior fracture and femoral neck BMD, these associations were attenuated (Table 2 and Fig. 2b).

**Table 2:** Associations between fracture and use of angiotensin converting enzyme inhibitors (ACEI) or angiotensin receptor blockers (ARB)

Men	Non-users without hypertension (*N* = 239)	Non-users with hypertension (*N* = 356)	*p* value	ACEI (*N* = 197)	*p* value	ARB (*N* = 107)	*p* value
Unadjusted	Referent	1.54 (1.00–2.37)	0.049	1.90 (1.18–3.05)	0.008	2.15 (1.26–3.66)	0.005
Adjusted^a^	Referent	1.06 (0.68–1.66)	0.799	1.05 (0.63–1.76)	0.859	1.46 (0.83–2.56)	0.184

Women	Non-users without hypertension (*N* = 220)	Non-users with hypertension (*N* = 162)	*p* value	ACEI (*N* = 76)	*p* value	ARB (*N* = 116)	*p* value
Unadjusted	Referent	1.13 (0.73–1.74)	0.588	1.74 (1.07–2.83)	0.025	1.12 (0.70–1.80)	0.641
Adjusted^b^	Referent	0.88 (0.54–1.43)	0.596	1.28 (0.74–2.22)	0.375	0.92 (0.53–1.57)	0.750

Data presented as hazard ratios and 95% CIs

^a^Adjusted model includes age, prior fracture, femoral neck bone mineral density and ARB dose

^b^Adjusted model includes age, alcohol consumption, prior fracture and femoral neck bone mineral density

However, the point estimate for fracture risk in men who used ARB medications remained elevated compared to the other groups. Additionally, there was evidence that the dose of ARB medication was important; the DDDs for men who did and did not sustain an incident fracture were 1.0 and 2.0, respectively. Therefore, an additional analysis was conducted, comparing ‘high’ and ‘low’ doses of ARB medication (high; *N* = 56, low; *N* = 40, missing; *N* = 11).

Compared to men taking a high dose of ARB medication, men taking a lower dose had a lower weight (mean ± SD; 82.4 ± 11.8 vs 90.4 ± 16.3 kg, *p* = 0.047) and higher number of comorbidities (median(IQR) Charlson Comorbidity Score; 1 (0–1), vs 0 (0–1), *p* = 0.05). The proportions of men who sustained an incident fracture over the follow-up period for high and low dose ARB use were 8.7% and 27.5%, respectively. In unadjusted analyses (Table 3), compared to non-users without hypertension, men taking ACEIs (HR 1.87, 95% CI 1.16–3.01, *p* = 0.010) and low dose ARBs (HR 2.66, 95%CI 1.34–5.29, *p* = 0.005) had a higher risk of incident fracture. Non-users of ACEI/ARB medication with hypertension also had an elevated risk, however, this did not reach significance (HR 1.53, 95% CI 0.99–2.35, *p* = 0.054). Men using higher dose ARBs did not have an elevated risk of incident fracture (HR 0.71, 95%CI 0.17–2.98, *p* = 0.643). In analyses adjusted for age, prior fracture and femoral neck BMD (Table 3), the association with low dose ARBs was sustained (HR 2.03, 95%CI 1.01–4.08, *p* = 0.048). The association for non-users with hypertension (HR 1.04, 95%CI 0.66–1.62, *p* = 0.879) and ACEIs was attenuated (HR 1.00, 95%CI 0.60–1.68, *p* = 0.987). The association for men taking a higher dose of ARB medication also remained non-significant (HR 0.27, 95%CI 0.04–1.99, *p* = 0.199).

**Table 3:** Analyses for men with angiotensin receptor blocker (ARB) use categorised as “low” (0 < defined daily dose ≤ 1) and “high” (> 1). Data presented as hazard ratios and 95% CIs

Men	Non-users without hypertension (*N* = 239)	Non-users with hypertension (*N* = 356)	*p* value	ACEI (*N* = 197)	*p* value	ARB low dose (*N* = 40)	*p* value	ARB high dose (*N* = 56)	*p* value
Unadjusted	Referent	1.53 (0.99–2.35)	0.054	1.87 (1.16–3.01)	0.010	2.66 (1.34–5.29)	0.005	0.71 (0.17–2.98)	0.643
Adjusted^a^	Referent	1.04 (0.66–1.62)	0.879	1.00 (0.60–1.68)	0.987	2.03 (1.01–4.08)	0.048	0.27 (0.04–1.99)	0.199

^a^Adjusted model includes age, prior fracture and femoral neck bone mineral density

ACEI = angiotensin converting enzyme inhibitor

11 men missing information on ARB dosage

#### Women

Of the 574 women, 76 (13.2%) used an ACEI medication, 116 (20.2%) used an ARB medication and 382 (66.6%) used neither an ACEI nor ARB medication (“non-users”). Within the non-users group, there were 162 women with hypertension and 220 without hypertension.

### Descriptive characteristics

Compared to non-users without hypertension, women in the other three groups were older, shorter, had higher BMI, higher systolic blood pressure, were more likely to have lower physical activity and had a higher Charlson Comorbidity Index Score (Table 1). Women in the non-user with hypertension and ARB user groups had a higher weight and diastolic blood pressure compared to women who were in the non-user without hypertension group. Additionally, compared to non-users without hypertension, women in the non-user with hypertension and ACEI user groups had lower BMD, however, women with ARB did not have a lower BMD. Women taking ACEI or ARB medications were more likely to use a statin than women in the non-user without hypertension group, whereas women taking an ACEI medication were more likely to use a medication that positively affects bone than the other three groups. Thiazide diuretic use was also higher in women taking ACEI or ARB medications than in the other two groups.

### Incident fracture risk

During 5156 person-years of follow-up (median, IQR: 10.9, 6.3–11.6 years), 135 women sustained at least one fracture. This was a rate of 2.62 (95% CI 2.21–3.10) fractures per 100,000 person-years of follow-up. These included 41 vertebra, 26 forearm/wrist, 11 ankle, 10 foot, 10 hip, 10 humerus, 10 rib, 4 pelvis, 3 femur, 3 patella, 3 tibia/fibula, 2 hand, 1 clavicle and 1 scapula fracture. The duration of follow-up for those with and without fracture were (median, IQR) 3.9 years (1.9–6.4) and 11.3 years (10.5–11.8), respectively.

The Kaplan–Meier plot for incident fracture risk is shown in Fig. 2c. Compared to non-users without hypertension, women who used ACEIs had a higher risk of incident fracture in unadjusted analyses (Table 2). Following adjustment for age, alcohol consumption, prior fracture and femoral neck BMD, this association was attenuated (Table 2 and Fig. 2d). No other differences between the groups were observed. There was no evidence of a dose effect for ARB medication use; the proportions of women who sustained an incident fracture during the follow-up period were similar for low and high dose (22.7% vs 19.4%, *p* = 0.726).

## Discussion

This study detected no associations between fracture risk and ACEI or ARB use compared to non-users without hypertension in women. However, men using a lower dose of ARB medication had a higher risk of fracture than non-users without hypertension. Men with hypertension who did not use ARBs or ACEIs were also at an increased risk for fracture but this did not reach significance. This result is not unexpected, as previous literature suggests an increased risk of fracture in the presence of hypertension [4, 35]. No other associations were detected for men. Thiazide use did not alter the relationships observed in either men or women.

Previous studies have performed similar analyses with differing results. Several studies have utilised administrative databases such as a study by Choi et al. [10], which followed male and female participants over a 1.9 year period using medical claims data. The study reported that ARB use was not associated with an increased risk of fracture compared to non-users (HR, 95%CI 1.00, 0.95–1.05). However, ACEI users were at an elevated risk (1.68, 1.49–1.91). Models were adjusted for age, sex, diabetes and osteoporosis (diagnosis and treatment), however, data were not available for BMD or clinical measurements such as weight and height. In another study, Medicare data from the USA were used to follow new users of antihypertensive medications for 60–90 days [13]. Compared to calcium channel blocker use (referent group), men and women using ARB medications had a lower risk of fracture (HR, 95%CI 0.76, 0.68–0.86) whereas those using ACEI medications showed no difference (0.96, 0.90–1.04). The models in these analyses were adjusted for age, sex, ethnicity, osteoporosis (diagnosis and treatment), prior fracture, BMD, other medications, falls, hospitalisations and comorbidities. Importantly, height and weight were not included in these models. Additionally, the eligibility criteria for Medicare (age ≥ 65 years, USA citizen ≥ 5 years, younger person with a disability and people with end stage renal disease) could have resulted in a sample that was not generalisable to other populations. Kao et al. [15] used a health insurance database to follow participants with hypertension over a six year period. The results showed that both ACEI and ARB use were associated with a lower risk of fracture compared to non-users (HR, 95%CI 0.70, 0.62–0.79 and 0.58, 0.51–0.65, respectively). Potential confounders considered in the models included age, sex, comorbidities, socioeconomic variables and other medication use, however, weight and BMD were not available.

In a cross-sectional study, Rejnmark et al. [17] used data from computerised registers to investigate the risk of fracture in the year 2000, with the independent variable being ACEI use in the past five years. The study showed that compared to non-users, ACEI use was associated with a reduced risk of fracture (OR 95% CI, 0.93, 0.90–0.96). The models were adjusted for prior fracture, comorbidities, other medication use, hospitalisations, employment status and socioeconomic factors. Age and sex stratified analyses were also completed, which showed similar results. Another study examined the risk of fracture in new ACEI users compared to new ARB users using administrative databases [14]. The participants were aged > 65 years and time to fracture was similar for both ACEI and ARB users (mean ± SD; 1.7 ± 1.7 and 1.9 ± 1.8 years, respectively). The study reported that compared to ACEI users, ARB users did not have an increased risk of osteoporotic fractures (HR, 95%CI 0.90, 0.74–1.08). In this study, the models were adjusted for medication dose, though it is not clear whether the other available variables were included in the models.

Bokrantz et al. [11] investigated the association between hip fractures and ACEI or ARB use in hypertensive men and women aged ≥ 50 years using data from the Swedish Primary Care Cardiovascular Database. Participants were followed for a maximum of six years. The results showed that compared to non-users with hypertension, there were no differences in risk of hip fracture for either ACEI (HR, 95%CI 1.05, 0.95–1.15) or ARB use (0.98, 0.87–1.11). Models were adjusted for age, sex, BMI, smoking status, prior fracture, diabetes, other medications, comorbidities and socioeconomic variables. However, smoking and BMI data were missing for many participants in this study. The results were similar for men and women separately. An additional study by Ruths et al. [18] used data from the Norwegian Prescription Database and Norwegian Hip Fracture Registry to investigate the association between antihypertensive medications and hip fracture over a six year period. The authors reported an age effect, where individuals aged < 80 years using ACEI medication had an increased risk of hip fracture, while those aged ≥ 80 years had a reduced risk of hip fracture. Hip fracture risk was also reduced for individuals taking ARB medications.

A few cohort studies have also been used to investigate the risk of fracture in users of ACEI and ARB medications. A study by Kwok et al. [12] compared ACEI and ARB use to non-users with hypertension using data from the Osteoporotic Fractures in Men Study (MrOS), which followed 2573 men aged ≥ 65 years for a mean of 6.8 years. The results showed that compared to non-users, men taking ACEI or ARB medications had a lower risk of fracture. The authors also reported that a higher duration of ARB use was associated with a larger reduction in fracture risk, however, the same was not observed for ACEI use. Models were adjusted for age, other medications, prior fracture, inability to complete a narrow walking trial, falls, BMD and depressed mood. This study did have data on weight, but this was not included in the final models. Another study followed women over a median 6.5 year period and showed that ACEI use was not associated with fracture risk (HR, 95%CI 0.78, 0.47–1.29) and neither was ARB use (1.16, 0.69–1.98) [36]. However, the authors reported that there was a dose effect observed, where longer term use (> 3 years) was associated with a lower risk of fracture, whereas the opposite was true for shorter term use (≤ 3 years). The models were adjusted for a large number of potential confounding variables including age, BMI, ethnicity, smoking status, alcohol consumption, prior fracture (self and parents), comorbidities and other medication use.

Other studies have investigated the risk of fracture considering ACEIs and ARBs combined, rather than separately. For example, Chen et al. [37] used a health insurance database to investigate the risk of fracture for men and women with hypertension who were aged ≥ 40 years. The results showed that individuals using a RAAS blocker (which included ACEIs, ARBs and mineralocorticoid receptor antagonists) had a lower risk of fracture compared to non-users (HR, 95%CI 0.66, 0.59–0.75). This effect was observed for both men and women, as well as across multiple age groups. The models were adjusted for age, sex, comorbidities, other medications and socioeconomic factors, however, weight and BMD were not available. Another study by Shea et al. [16] included men and women aged ≥ 65 years and determined the risk of hip fracture for users of ACEIs or ARBs (combined). Compared to non-users, those who used ACEI/ARB medications had a lower risk of hip fracture (HR 95%CI, 0.707, 0.585–0.853). The models were adjusted for age, sex, socioeconomic variables, prior fracture and other medication use. Kunutsor et al. [38] conducted both a meta-analysis and a cohort study in their publication, the latter of which followed men and women for a median of 14.8 (12.8–15.8) years. The authors reported that ACEI or ARB use was not associated with fractures compared to non-users (HR, 95%CI 1.00, 0.59–1.69). Their models included age, sex, BMI, smoking status, alcohol consumption, diabetes, blood pressure, other medication use, socioeconomic status and physical activity. In these three above studies, it is not clear if the associations are driven by ACEI use, ARB use, or both, as other studies have reported that the two different classes of medication have differing associations with fracture.

Overall, there have been several studies that have examined associations between ACEI and/or ARB medication use and fracture risk. However, the results have been inconsistent and this may be due to heterogeneity among studies, making meaningful comparisons difficult. We have described examples of this heterogeneity above, highlighting the major differences in studies that make it challenging to compare our data with previous work. For example, many studies did not have data for potential confounding variables such as weight and BMD, some had short or unspecified follow-up duration, some examined hip fractures only, some include men and women whereas others only include one sex, some have combined ACEIs and ARBs whereas others have not, different studies have used different control groups, some studies have examined new users of ACEIs and ARBs whereas others included longer duration users and age groups differ across the studies. Our study has some strengths in that we have examined ACEI and ARB use separately, both men and women were included, adjustments for other confounding variables were made (particularly weight and BMD), follow-up time was long and all fractures were considered (except high trauma, face/skull, fingers and toes).

Our results for ACEI use do agree with several studies reporting no association with fracture risk [11, 13, 36]. However, there are also studies reporting an elevated fracture risk [10] or lower fracture risk [12, 15, 17]. Different associations have also been reported by age, where fracture risk was both elevated and reduced depending upon age (Ruths et al. [18]). In several studies, no associations between ACEI use were observed when younger age groups were considered (e.g. ≥ 50 years) [11, 36] (and this study), whereas two studies including an older group of participants (e.g. ≥ 65 years) have reported differences in fracture risk [12, 17]. Overall, for ACEI use, previous literature, as well as our own study generally indicate a lower or unaffected risk of subsequent fracture. No previous studies have reported that ARB use was associated with a higher risk of fracture in men. Previous work has generally indicated a reduction in fracture risk with ARB use, irrespective of age group studied [12, 13, 15, 18]. It is unclear why our study differs from the literature, but may be related to ARB medications not being available in Australia until 1997, whereas ACEI medications were available much earlier [27]. Of the men in this study who were currently taking an ARB medication, 29 reported taking another antihypertensive medication previously. Of these, 10 were previously taking an ACEI medication, five were taking an ARB medication and 14 were taking a different type of antihypertensive medication (such as a calcium channel blocker). For men currently taking an ACEI medication, 19 had a previous antihypertensive medication, where 12 of these were on previous ACEI medication, while the other seven were neither ACEI nor ARB medications. None of the current ACEI users had previously taken an ARB medication. It is possible that some individuals currently taking ARB medication may have changed from a previous antihypertensive medication due to insufficient reduction in blood pressure, perhaps with a background of other comorbidities, or alternatively experienced adverse drug events such as persistent cough. In this study, men taking a lower dose of ARB medications had a higher number of comorbidities compared to those taking a higher dose, which may have affected fracture risk. One comorbidity, diabetes, which is known to affect fracture risk [39–41], was more common among those using low dose (17.1%) compared to high dose (8.3%) ARB medications. Men taking a lower dose of ARB medication also had lower weight compared to those taking a higher dose and this is important because BMD increases with increasing BMI [42]. Femoral neck BMD appeared to be lower for men taking lower compared to higher ARB doses, though the difference was not significant (*p* = 0.699). Another possible reason for the results observed in this study is that individuals taking a lower dose of ARB medications may not have sufficient blockage of the angiotensin II receptor type 1. Since angiotensin II is detrimental to bone by promoting bone resorption and inhibiting bone formation [8, 9], it is possible that insufficient blockade of the angiotensin II receptor may lead to reduced bone quantity and quality leading to an increased fracture risk. Finally, the men currently using an ARB medication may also have had specific unknown characteristics that placed them at a higher risk for fracture that were not accounted for in the analyses.

In this study, we also reported different results for men and women, which may be related to differences in response to RAAS inhibition. It has been reported that female rats had a greater change in blood pressure compared to males following administration of the ACEI, enalapril [24]. It has also been reported that overall, males have greater expression levels and responses to angiotensin II, angiotensin II type 1 receptor and the angiotensin converting enzyme compared to females [22]. Additionally, androgens can promote the synthesis of angiotensinogen, which leads to higher levels of angiotensin II [25]. Therefore, it is possible that men in this study showed a greater response to angiotensin II levels, leading to different observations compared to women, who may have had a greater response to RAAS inhibition. Additionally, participants in this study were aged ≥ 50 years, where the fracture risk for women is higher than in men, following menopause [43]. Thus, it is also possible that women in this study were at an increased risk for fracture, such that RAAS inhibition was not a significant risk factor for fracture, while for men this was not the case.

While the assessment of other anti-hypertensive agents and their association with fracture risk was outside the scope of this study, we note that previous literature shows mixed associations between other anti-hypertensives (such as loop diuretics, thiazides and beta blockers) and fracture risk [11, 13]. This may be an avenue for future research.

There were several strengths and limitations to this study. The participants were randomly selected and population-based. Some self-reported data were used in this study, however some variables including fracture endpoints were objectively measured. However, since radiological reports were used to determine fracture endpoints, there was minimal loss to follow-up, even if a participant did not attend a subsequent follow-up visit. In determining fractures, we have taken an inclusive approach and utilised ICD-9 codes for cause of injury which aim to capture all minimal trauma fractures, and acknowledge that we cannot guarantee that all higher trauma fractures were excluded from the study. National Deaths Index data were also used to determine date of death, which further minimised bias from loss to follow-up. Non-users without hypertension were younger than their counterparts with hypertension, therefore, models were adjusted for age, but additional confounding from other factors associated with age may not have been captured in these models. Additionally, data were available for other potential confounders including weight, other medications and comorbidities. However, these confounders and medication use were assessed at a single time point prior to assessment of fracture and changes may have occurred in these variables in the intervening time period. There may also be residual confounding that had not been taken into account. Additionally, since medication use was only assessed at a single time point, and this was earlier for men than women, it is possible that changes in the prescription of ARB medications in Australia over time could have influenced the observed differences between the sexes. It is also possible that this study had insufficient power to detect differences between the groups, particularly for women. An association between ACEI use and fracture risk was observed in unadjusted analyses, though this was attenuated following adjustment for other variables. However, the point estimate remained elevated in adjusted analyses and it is possible that there was insufficient power to detect any differences. There was also an insufficient number of participants using different types of ACEI and ARB medications (e.g. irbesartan and candesartan for ARBs) and it was not possible to assess differences between these different agents. In addition, compliance with medication dose was not available. It is also possible that some participants changed medication dose or type during the follow-up period and this could have affected the results.

## Conclusions

For women, ACEI or ARB use was not associated with increased risk of incident fracture. In men, a dose effect was observed, where men taking a lower dose ARB medication were more likely to sustain an incident fracture. This study adds to the literature regarding the effect of ACEI and ARB use on fracture risk, however, additional studies with larger sample sizes and longer follow-up times are needed to confirm these findings.
